# Socioeconomic differences in the intensity of end-of-life care among patients with cancer: a population-based study in the Lazio region, Italy

**DOI:** 10.1093/eurpub/ckag148

**Published:** 2026-08-17

**Authors:** Arianna Bellini, Alessandro Cesare Rosa, Michela Servadio, Paola Michelozzi, Antonio Addis, Valeria Belleudi

**Affiliations:** Department of Epidemiology, Lazio Regional Health Service, Rome, Italy; Department of Epidemiology, Lazio Regional Health Service, Rome, Italy; Department of Epidemiology, Lazio Regional Health Service, Rome, Italy; Department of Epidemiology, Lazio Regional Health Service, Rome, Italy; Department of Epidemiology, Lazio Regional Health Service, Rome, Italy; Department of Epidemiology, Lazio Regional Health Service, Rome, Italy

## Abstract

The importance of assessing end-of-life (EoL) care has grown due to increased life expectancy and chronic disease prevalence. Despite awareness of holistic care, cancer patients often receive aggressive treatments that may reflect disparities influenced by socioeconomic factors. This study investigates EoL care among cancer patients in Italy, focusing on the impact of socioeconomic position (SEP). A retrospective observational study was conducted using Italian health databases, focusing on cancer patients aged 35 years and older who died between 2015 and 2019 in Lazio, Italy. SEP was assessed using educational attainment and a composite deprivation indicator at the census-section level. EoL care intensity was measured using hospitalization rates, emergency department visits, medication use, and place of death. Logistic regression models [OR (95% CI)] were used to explore socioeconomic associations with care intensity. The study included 84 936 subjects, predominantly aged over 75, with lung, colorectal, and breast cancers being the most common. During the last month of life, individuals with middle to higher educational levels (upper-middle, university degrees) had increased risks of hospitalization (OR 1.06; 95% CI: 1.02–1.10; OR 1.09; 95% CI: 1.03–1.15), anti-neoplastic therapies (OR 1.29; 95% CI: 1.21–1.38; OR 1.48; 95% CI: 1.36–1.61), and in-hospital death (OR 1.08; 95% CI: 1.03–1.12; OR 1.16; 95% CI: 1.10–1.23) and lower risks of ED admissions (OR 0.91; 95% CI: 0.87–0.94; OR 0.76; 95% CI: 0.71–0.80) and opioid therapy (OR 0.90; 95% CI: 0.86–0.94; OR 0.87; 95% CI: 0.82–0.92). Similar results were observed when using the area-based SEP indicator. Socioeconomic differences influence EoL care pathways for patients with cancer in Italy. These findings suggest complex interactions between socioeconomic factors and care preferences, highlighting the need for tailored and equitable EoL care strategies.

Key points

- Socioeconomic position may influence the intensity and quality of end-of-life care among patients with cancer, but evidence on these inequalities in Italy is still limited.
- We conducted a retrospective population-based study of 84 936 Lazio Region residents aged 35 years or older who died from cancer between 2015 and 2019, using linked administrative healthcare data to assess indicators of end-of-life care intensity.
- In the last 30 days of life, patients with lower socioeconomic position showed greater emergency department use, lower use of hospital-based and antineoplastic care, lower probability of in-hospital death, and higher outpatient opioid prescribing.
- Evaluating end-of-life care indicators over time and across healthcare settings may help identify socioeconomic disparities and support more equitable and integrated care strategies, including actions to reduce emergency department use among lower-SEP patients and assess potential overtreatment or inefficiencies related to antineoplastic treatment near death.

## Introduction

Population aging and rising burden of non-communicable diseases pose major challenges for healthcare systems in ensuring adequate end-of-life (EoL) care [1]. The OECD’s 2023 report, Time for Better Care at the End of Life [2], estimated that nearly 90% of deaths in OECD countries between 2001 and 2019 were attributable to conditions potentially amenable to EoL care, with cancer accounting for approximately one quarter of these deaths. The same report projects that the number of people in need of EoL care will increase from 7 million in 2019 to nearly 10 million by 2050, underscoring its growing relevance as a global public health priority.

Cancer care represents a key challenge in this context. Advances in diagnosis and treatment have prolonged survival, transforming many cancers into chronic progressive conditions. As a result, more patients live longer with advanced disease and eventually dying from it, making the EoL phase an increasingly important component of oncology care. At this stage, care should shift from disease-directed and life-prolonging interventions toward a more comprehensive, patient-centered approach focused on symptom control, quality of life, and alignment with patients’ values and preferences [3–5]. From this perspective, the early integration of palliative care into oncology pathways has been associated with improved outcomes and reduced unnecessary healthcare utilization [6, 7]. Despite this shift in recommended models of care, studies continue to highlight a predominantly hospital-centred approach to EoL care, characterized by high rates of hospitalization, administration of anticancer treatments near death, and late referral to palliative care services [8, 9]. Such interventions in the last weeks or months of life are widely considered markers of poor-quality care. Evidence from international and Italian studies shows that these care patterns remain common among patients with cancer, highlighting a persistent gap between recommended care models and real-world clinical practice [10–13].

It is well known that health follows a social gradient, and that socioeconomic position (SEP) can influence health outcomes through a series of biological, physiological, and environmental mechanisms [14–16].

In recent years, evidence has shown that the social gradient in health persists even in the last year of life. SEP may influence EoL care through at least three interrelated pathways: health and functional status, access to healthcare services, and social support [17]. Individuals with lower SEP often experience a higher burden of illness and disability, increasing care needs and the likelihood of hospital-based care. At the same time, they may face greater barriers to accessing elective, primary, and social care services due to lower health literacy, limited awareness of available EoL services, financial constraints, or practical obstacles such as transportation [18]. Finally, weaker informal care networks and unsuitable housing conditions may reduce the feasibility of home-based care, making hospitalization more likely in the final months of life. Together, these mechanisms help explain how socioeconomic disadvantage shapes patterns of care and service utilization at the EoL.

Building on these pathways, the way SEP is operationalized is also relevant. As Davies *et al.* [19] pointed out, different measures of SEP may capture distinct underlying mechanisms. For instance, education is likely to primarily reflect health literacy and awareness of available services, thus relating more closely to patients’ ability to navigate the healthcare system. In contrast, measures of deprivation [20] may better capture structural and material constraints, including housing conditions, family context, and broader socioeconomic environment.

Despite growing international evidence on socioeconomic inequalities in EoL, data on the intensity of care delivered to patients with cancer in Italy remain limited. Few population-based studies have examined patterns of potentially aggressive or hospital-centred care in the last months of life [13, 21, 22], and, to our knowledge, evidence on whether these patterns vary according to patients’ SEP is still lacking. This represents an important knowledge gap in a healthcare system such as the Italian National Health Service, which is founded on principles of universal and equitable access but may still reproduce inequalities in the delivery and utilization of care.

In this context, the aim of our study was to evaluate indicators related to the intensity and quality of EoL care among patients with cancer in the Lazio Region and to investigate potential differences according to socioeconomic factors during the last phase of life.

## Methods

Our study has a retrospective observational design and involved individuals who died with a diagnosis of cancer between 2015 and 2019 in the Lazio Region. Italy has a National Healthcare System providing universal coverage, largely free of charge, and organized on a regional basis. Lazio is a large region in central Italy with approximately 5.7 million inhabitants and 10 Local Health Authorities (LHAs). The study considered the following regional administrative healthcare claims:

- The hospital information system, which collected data from all regional hospitals, including patient demographics, admission referral source, discharge status, up to six discharge diagnoses, and up to six hospital procedures.
- The healthcare assistance registry (approximately 97% of residents), which included information about the date of birth, gender, date of registration in the regional healthcare system, and, where applicable, the date of death or deregistration.
- The healthcare emergency information system, which collected information on Emergency Department (ED) visits.
- The regional drug-dispensing registry, which included records for each medical prescription dispensed in public and private pharmacies, as well as chemotherapy treatments supplied by hospital pharmacies at discharge.
- The outpatient diagnostic tests and specialist outpatient visit database, which included test or visit codes, test dates, and the names of laboratories where the tests were performed.
- The Nominative Registry of Causes of Death (ReNCaM).

All regional health information systems included a unique person-based identifier, enabling deterministic record-linkage procedures.

### Study population

The study cohort was identified through ReNCaM and included individuals who died from cancer, identified using the “International Classification of Diseases 9th Revision Clinical Modification” (ICD-9CM) or the “International Statistical Classification of Diseases and Related Health Problems, 10th Revision” (ICD-10). The following codes were used for cohort selection: ICD-9CM 140-208; ICD-10 C00-C97.

The inclusion criteria required that individuals had died between 1 January 2015 and 31 December 2019, were residents of the Lazio Region at the time of death, received a diagnosis of cancer and were aged 35 years or older. The choice of the age criterion was based on the decision to use educational level as a measure of SEP, as discussed above. This required the inclusion of subjects aged 35 years or older, in order to ensure the availability of a reliable indicator of their highest attained level of education.

The baseline characteristics of the patients included in the analysis encompassed sociodemographic variables such as age, gender, and LHA of residence. Charlson Comorbidity Index (CCI), a validated measure of comorbidity burden, was also considered [23]. Additionally, data on tumor site were collected to further characterize the underlying oncological conditions.

### Socioeconomic position

Educational level was used as the first measure of SEP and was categorized into four ordinal levels: low (no formal education/primary school), low-medium (lower secondary school), medium-high (upper secondary school), and high (university degree or higher). Educational level has been widely used as an individual indicator for SEP [24], as it serves as a proxy for people’s cognitive resources, ability to process health information, and is associated with future income and occupation [25]. All the analyses were replicated using an area-based SEP index [20]. This national deprivation index, calculated at the census section level based on 2011 census data, considered education, occupation, housing tenure, family composition, living in a crowded house, and living in a single-parent family. Based on information about the patient’s residence at the time of death, each patient was assigned to a census section through a record linkage procedure and consequently to a SEP quintile (1 = least deprived; 5 = most deprived).

### End-of-life care quality measures

Based on the literature, we defined a set of indicators covering key dimensions of EoL care. Earle *et al.* [8] first developed performance measures for EoL cancer care through a literature review and focus groups. These indicators were subsequently validated and benchmarked [26] and later tested and adopted in different countries [9, 27, 28]. Some of the selected measures describe the use of highly intensive treatments and hospital-based services near death, whereas others provide information on symptom management and the setting in which care is delivered.

Operationally, we measured the proportion of patients who experienced at least one hospitalization, at least one ED visit, or at least 1 day in the intensive care unit (ICU) as well as the proportion who received antineoplastic therapies (chemotherapy and/or immunotherapy), radiotherapy, or life-sustaining surgical interventions. Among patients with at least one ED visit in the last 30 days of life, we also calculated the proportion of ED visits resulting in hospitalization. An additional indicator was the proportion of patients who received opioid treatment, given the importance of pain management in EoL care [10, 29]. Finally, the place of death was considered, as many patients express a preference for dying at home [30]. All indicators were assessed at 14, 30, 60, and 90 days before death. The ICD-9-CM and ATC codes used to identify these indicators within healthcare administrative databases are reported in Supplementary Table S1.

These measures should not be interpreted as direct measures of care appropriateness, as EoL care is shaped by clinical circumstances as well as by patients’ preferences, values, beliefs, and available support. Their interpretation therefore requires caution, particularly for measures such as opioid treatment and place of death, whose meaning may vary substantially across individual and organizational contexts. Nevertheless, when examined at the population level, these indicators can help identify relevant variations in care and areas warranting further investigation [8].

### Data analyses

Descriptive analyses of the demographic and clinical characteristics of the cohort used frequencies and proportions for categorical variables and means with standard deviations, or medians with quartiles, for continuous variables. Patients were stratified by SEP, using educational level and the area-based SEP indicator as exposure measures. Differences between groups were assessed using *t*-tests for continuous variables and chi-square (χ²) tests for categorical variables. The associations between each EoL care indicator measured during the last 30 days of life and educational level were assessed using logistic regression models. The adjustment variables included age, sex, CCI, LHA of residence, and tumor site. The analyses were then replicated using the area-based SEP index. Patients with missing data on educational level or the area-based SEP indicator were excluded from the corresponding analyses.

## Results

Supplementary Figure S1 shows the cohort selection process. Overall, 298 699 individuals were recorded in the ReNCaM between 1 January 2015 and 31 December 2019. Among them, 88 077 died from neoplastic diseases; after restricting the sample to individuals residing in the Lazio Region at the time of death and aged 35 years or older, the final study cohort included 84 936 individuals.

Among the 84 936 individuals included in the study, most were aged 75 years or older at the time of death (57.4%), and approximately half resided in the three most populous Local Health Authorities (LHAs) within the metropolitan area of Rome (Table 1). The most frequent tumor sites were lung (22.1%), lymphatic and hematopoietic tissue (17.7%), colon (11.4%), pancreas (6.8%), and breast (6.7%); this distribution was broadly consistent across educational groups, although lung cancer was relatively more common among individuals with lower secondary or upper secondary education, whereas breast cancer was more frequent among those with higher educational attainment.

**Table 1:** Characteristics of the cohort overall and by educational level.^a^

	Total cohort		Education level			
	*N*	%	No formal education/primary school	Lower secondary school	Upper secondary school	University degree or higher
Overall (*n*)	84 936		34 662	20 565	19 080	7536
Sex						
M	46 458	54.7%	49.9%	61.1%	54.5%	61.3%
F	38 478	45.3%	50.1%	38.9%	45.5%	38.7%
Age						
35-64	16 042	18.9%	5.3%	26.2%	32.5%	26.9%
65-74	20 152	23.7%	18.5%	27.8%	27.7%	27.2%
75+	48 742	57.4%	76.2%	46%	39.8%	45.9%
Median	75		80	72	70	72
1 quartile-3 quartile	68-84		75-86	64-81	61-80	64-82
No. comorbidities						
0-1	23 098	27.2%	25.8%	22.1%	23.5%	27.1%
2	29 894	35.2%	31.2%	37.9%	42.5%	42.2%
3	16 641	19.6%	20.7%	21.0%	19.8%	18.2%
4+	15 303	18.0%	22.3%	19.0%	14.3%	12.5%
Tumor site						
Lung bronchi and trachea	18 804	22.1%	20.5%	24.5%	23.0%	21.1%
Upper gastrointestinal tract	4836	5.7%	6.7%	5.4%	4.6%	4.2%
Colon and rectum	9658	11.4%	12.1%	10.9%	10.8%	10.4%
Liver and biliary tract	4923	5.8%	5.9%	6.1%	5.4%	5.3%
Pancreas	5796	6.8%	6.4%	6.7%	7.4%	7.7%
Breast	5682	6.7%	6.1%	5.9%	8.1%	8.1%
Female genital system	3446	4.1%	4.1%	3.8%	4.3%	4.0%
Male genitourinary system	3736	4.4%	4.6%	4.0%	3.8%	5.9%
Kidney and urinary tract	5300	6.2%	6.6%	6.4%	5.7%	5.7%
Central nervous system	2795	3.3%	2.5%	3.3%	4.2%	4.3%
Other solid tumors	4935	5.8%	5.5%	6.0%	6.0%	6.0%
Lymphatic and hematopoietic tissue tumors	15 025	17.7%	19.1%	16.8%	16.7%	17.1%
LHA of residence						
LHA of Rome 1	15 791	18.6%	13.3%	16.9%	23.9%	39.8%
LHA of Rome 2	19 064	22.4%	21.1%	24.4%	25.2%	23.7%
LHA of Rome 3	8926	10.5%	9.1%	11.8%	12.6%	11.2%
LHA of Rome 4	4562	5.4%	5.2%	5.8%	5.3%	3.8%
LHA of Rome 5	6825	8.0%	9.2%	8.5%	6.5%	3.3%
LHA of Rome 6	7310	8.6%	8.5%	9.0%	8.0%	5.1%
LHA of Viterbo	5006	5.9%	7.3%	5.2%	4.0%	3.1%
LHA of Rieti	2475	2.9%	3.7%	2.5%	2.3%	1.6%
LHA of Latina	7668	9.0%	10.9%	8.3%	6.4%	4.4%
LHA of Frosinone	7241	8.5%	11.7%	7.5%	5.8%	4.0%

a A total of 3093 individuals had missing education level data.

Regarding socioeconomic position, 40.8% of the cohort had a low educational level (no formal education or primary school), whereas 9.9% held a university degree or higher. Lower educational attainment was strongly concentrated among older individuals: 76.2% of those in the lowest educational group were aged 75 years or older, compared with 45.9% among those with a university degree or higher. Conversely, younger individuals were more represented in the intermediate and highest educational groups.

Differences were also observed in comorbidity burden, with four or more comorbidities reported in 22.3% of individuals in the lowest educational group compared with 12.5% among those with a university degree or higher. Characteristics of the cohort stratified by area-based SEP index are reported in Supplementary Table S2.

Table 2 summaries EoL care indicators among the study cohort. Overall, 25.9% of individuals received antineoplastic treatment in the last 90 days of life, decreasing to 10.8% in the last 30 days and 5.0% in the last 14 days. Similarly, 9.9% of patients initiated a new antineoplastic treatment within the last 90 days of life, including 3.1% in the last 30 days and 1.4% in the last 14 days. Hospital-based care was common near the EoL: 64.6% of patients experienced at least one inpatient hospitalization in the last 90 days of life, and approximately half of the cohort (50.2%) in the last 30 days. Although the mean length of hospital stay decreased as the observation window narrowed, the mean duration of stay remained relatively long, averaging 13.2 days in the last 30 days of life and 8.5 days in the last 14 days. Emergency department (ED) use was also frequent, involving 60.3% of patients in the last 90 days and 37.7% in the last 30 days. Among those with at least one ED visit in the last 30 days of life, 32.4% were not subsequently hospitalized. Intensive interventions were less frequent: life-sustaining treatments or procedures were recorded in approximately 1% of patients in both the last 30 and 14 days of life. Similarly, radiotherapy use declined from 6.5% in the last 90 days to 1.4% in the last 14 days. Finally, more than one-third of the cohort (35.1%) died in hospital in the last 30 days of life.

**Table 2:** Indicators of intensity of care in the end-of-life phase among the study cohort.

Intensity of care in the end-of-life phase		Cohort (84 936)		
		*n*	%	Mean
Individuals receiving antineoplastic treatment	Last 14 days of life	4243	5.0	
	Last 30 days of life	9143	10.8	
	Last 60 days of life	16 991	20.0	
	Last 90 days of life	21 961	25.9	
Individuals receiving a *new* antineoplastic treatment^a^	Last 14 days of life	1188	1.4	
	Last 30 days of life	2624	3.1	
	Last 60 days of life	5585	6.6	
	Last 90 days of life	8436	9.9	
Individuals with at least 1 day of inpatient hospitalization	Last 14 days of life	34 852	41.0	8.5 days
	Last 30 days of life	42 664	50.2	13.2 days
	Last 60 days of life	50 539	59.5	17.5 days
	Last 90 days of life	54 834	64.6	19.7 days
Emergency Department (ED) visits	Last 14 days of life	19 558	23.0	
	Last 30 days of life	32 061	37.7	
	Last 60 days of life	45 125	53.1	
	Last 90 days of life	51 214	60.3	
Percentage of ED visits resulting in hospitalization	Last 30 days of life	24 870	77.6	
Individuals admitted to the intensive care unit (ICU)	Last 30 days of life	2260	2.7	
Individuals undergoing at least one life-sustaining treatments/procedures in the last 30 days of life	Last 14 days of life	816	1.0	
	Last 30 days of life	982	1.2	
	Last 60 days of life	1150	1.4	
	Last 90 days of life	1231	1.4	
Individuals receiving radiotherapy	Last 14 days of life	1201	1.4	
	Last 30 days of life	2343	2.8	
	Last 60 days of life	4119	4.8	
	Last 90 days of life	5537	6.5	
In hospital deaths		29 813	35.1	

a Antineoplastic treatments were defined as “new” when no chemotherapy administration was identified in administrative databases during the previous 3 months.

**Figure 1:**
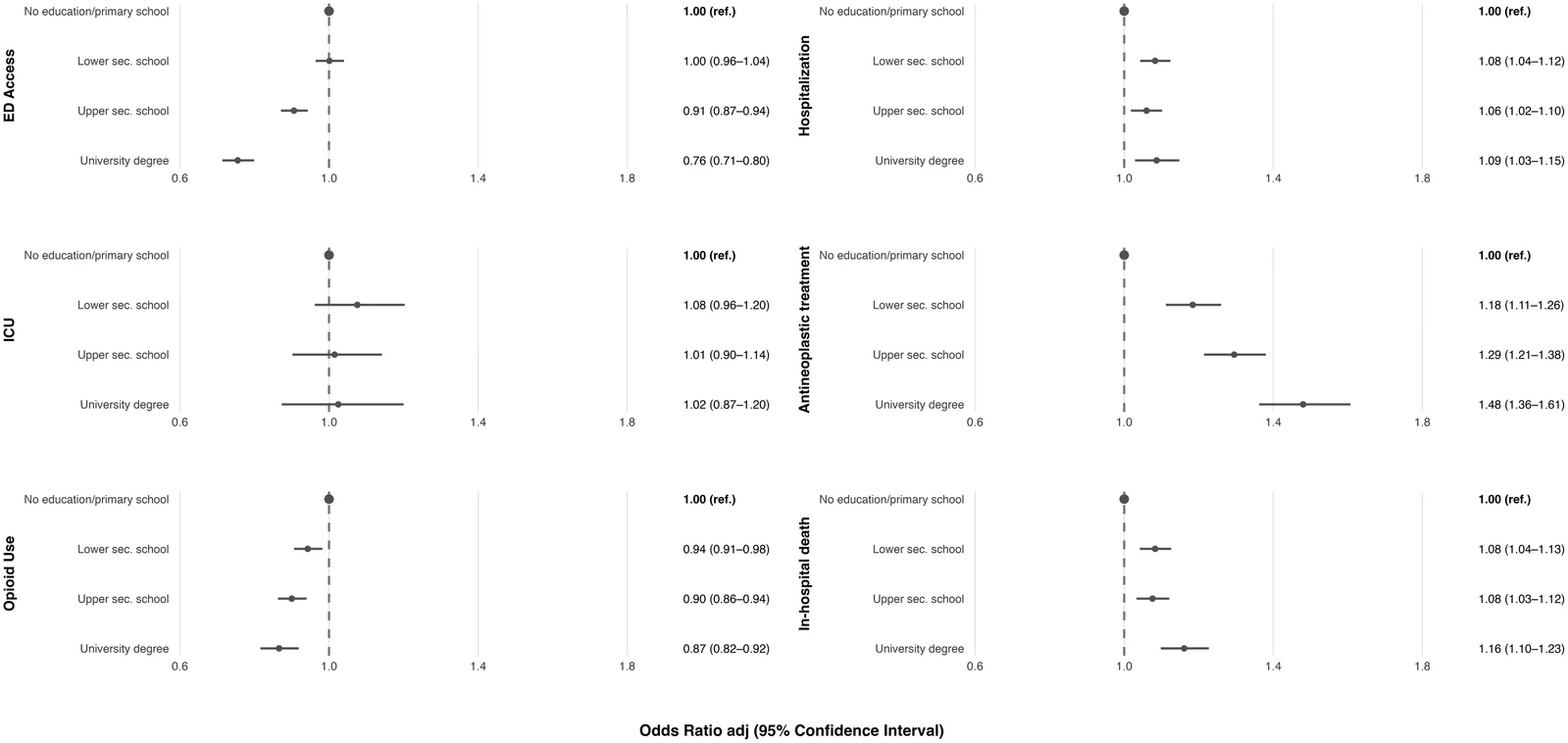
Adjusted odds ratios for end-of-life care indicators in the last 30 days of life according to educational level.

The results of the logistic regression model showed that, compared with individuals with low educational level, those with lower secondary, upper secondary, and university education had higher odds of hospital admission [OR (95% CI): 1.08 (1.04–1.12), 1.06 (1.02–1.10), and 1.09 (1.03–1.15)], receiving antineoplastic treatment in the last 30 days of life [OR (95% CI): 1.18 (1.11–1.26), 1.29 (1.21–1.38), and 1.48 (1.36–1.61)], and in-hospital death [OR (95% CI): 1.08 (1.04–1.13), 1.08 (1.03–1.12), and 1.16 (1.10–1.23)]. By contrast, individuals with upper secondary education and university degree or higher had lower odds of ED attendance [OR (95% CI): 0.91 (0.87–0.94) and 0.76 (0.71–0.80), respectively]. Moreover, higher educational level was associated with progressively lower odds of opioid use [OR (95% CI): 0.94 (0.91–0.98), 0.90 (0.86–0.94), and 0.87 (0.82–0.92)] (Fig. 1).

**Figure 2.**
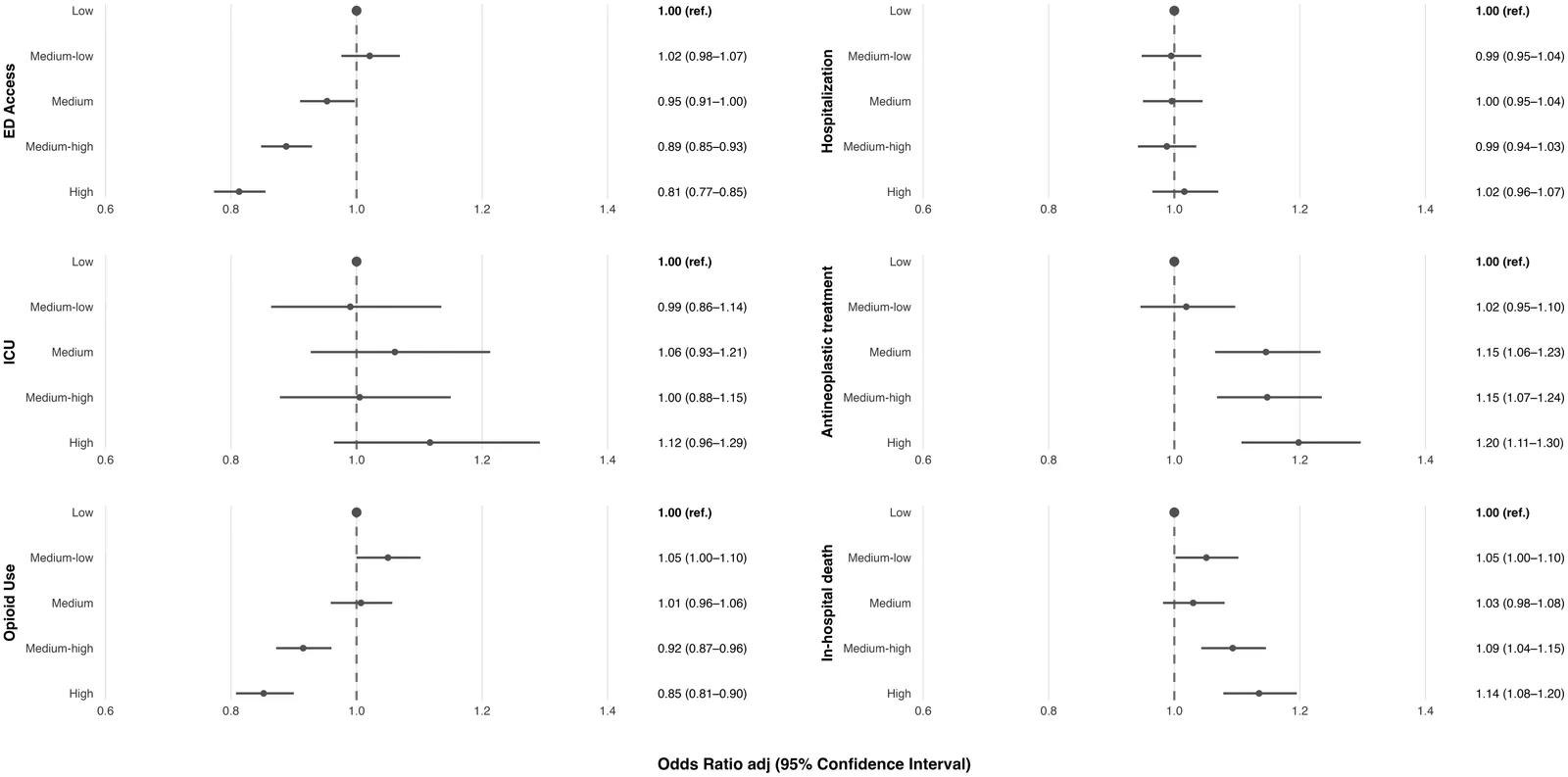
Adjusted odds ratios for end-of-life care indicators in the last 30 days of life according to area-based SEP index.

Similar patterns were observed when the area-based SEP index was used (Fig. 2), although associations were generally more heterogeneous across outcomes. In this case, no significant differences across different groups were observed for hospitalization or ICU admission, as estimates were close to the null value and confidence intervals generally included unity.

By contrast, the associations observed for ED access, in-hospital death, and the use of antineoplastic treatment and opioids were broadly confirmed. Compared with individuals living in the most deprived areas, those belonging to the two least deprived groups showed lower odds ED access [OR (95% CI): 0.89 (0.85–0.93) and 0.81 (0.77–0.85)], higher odds of in-hospital death [OR (95% CI): 1.09 (1.04–1.15) and 1.14 (1.08–1.20)], greater likelihood of receiving antineoplastic treatment [OR (95% CI): 1.15 (1.07–1.24) and 1.20 (1.11–1.30)], and lower odds of opioid use [OR (95% CI): 0.92 (0.87–0.96) and 0.85 (0.81–0.90)]. Overall, estimates were generally less precise than those obtained using educational level, particularly for intermediate area-based SEP categories.

## Discussion

The results of the present study highlight socioeconomic differences in EoL pathways: individuals with lower educational attainment appear to access ED more frequently, to be hospitalized less often, to die less frequently in hospital, and to make less use of antineoplastic treatment; conversely, they showed greater use of opioids. When the analyses were repeated using the area-based SEP index, consistent results were observed with respect to ED use, in-hospital death, and the use of antineoplastic treatment and opioids, although statistical significance was not reached for hospitalization.

Our findings related to ED attendance are in line with previous studies [31, 32], which showed greater reliance on emergency services toward the EoL among individuals with lower SEP. This pattern has been observed not only in non-universal healthcare contexts, such as the United States—where use of these services may be driven by lack of insurance or other financial barriers, but also in countries with universal healthcare systems. For example, a study conducted in England on patients with colorectal, breast, prostate, and lung cancer highlighted disparities in healthcare costs incurred by the system at the EoL, specifically associated with greater use of emergency care [33]. This greater reliance on emergency services among patients with lower SEP may be attributable to several factors: on the one hand, it may indicate limitations in the capacity of community-based services to provide adequate care [32, 34, 35]; on the other hand, it may reflect a higher symptom burden experienced by patients living in deprived conditions, together with related issues such as loss of autonomy or functional decline [36–38].

Conversely, the finding of greater use of hospital-based care among patients with higher SEP contrasts with recently published studies [19, 39, 40] reporting opposite results. Even though this finding seems to contradict expectations, it may in fact reflect greater demand for and/or provision of medicalized care among patients with higher SEP. A similar pattern was reported by Walsh and Laudicella [33] in the aforementioned study, in which patients with high SEP had a greater probability of elective hospital admission and a lower probability of emergency admission compared to those in the lowest SEP quintile. The authors hypothesized the existence of a substitution effect between elective and emergency admissions, whereby patients with lower SEP appear more likely to substitute emergency care for elective care.

Nevertheless, the higher proportion of patients hospitalized in the last 30 days of life in our study warrants cautious interpretation and should be considered alongside the greater use of antineoplastic treatment: in fact, although chemotherapy is often delivered in a day-hospital setting, in our data these episodes are classified as hospitalizations. Also, the finding of greater chemotherapy use among patients with higher SEP contrasts with the expectation that lower socioeconomic groups might receive more intensive treatment due to later-stage diagnoses. This pattern may reflect a combination of factors, including greater access to specialist oncology services, higher health literacy, and increased patient demand for aggressive treatments among individuals with higher SEP. The observed socioeconomic differences in opioid prescribing need to be considered in light of the data source used. The pharmaceutical registry captured only outpatient prescriptions reimbursed outside the hospital setting and did not include opioids administered during hospital admissions. Since patients with higher SEP spent more days in hospital during the last month of life, their overall opioid exposure may have been underestimated to a greater extent. Consequently, greater outpatient prescribing among lower socioeconomic groups may reflect differences in care setting rather than greater overall use of pain treatment. Other factors may also have contributed to this finding. As previously discussed, socially disadvantaged patients may have experienced a greater symptom burden and poorer baseline health conditions [36, 39], potentially resulting in a greater need for opioid treatment. These differences should therefore be interpreted as arising from multiple, potentially overlapping clinical and healthcare-related factors, rather than as direct evidence of more adequate pain management.

Place of death is widely considered a key indicator of EoL care, although its interpretation remains complex and highly context-dependent. Indeed, it reflects the interaction between individual preferences, family and caregiving resources, availability of community and palliative care services, and broader healthcare system organization [S41, S42]. Previous studies across several European countries have consistently shown that most individuals, particularly patients with advanced cancer, express a preference for dying at home rather than in hospital settings [30]. However, the ability to achieve the preferred place of death is socially patterned, with disadvantaged individuals often facing greater barriers [40]. In our study, information on patients’ preferences and on the availability and adequacy of home-based palliative care were not available. Therefore, the lower probability of in-hospital death observed among socioeconomically disadvantaged patients should not necessarily be interpreted as evidence of more patient-centered or preference-concordant care. It may instead reflect differences in access to and organization of EoL services, including more limited access to elective and specialist hospital-based care and a greater reliance on less planned or less integrated care pathways. This interpretation should be considered alongside the greater use of emergency services and lower use of antineoplastic treatments among disadvantaged patients.

To the best of our knowledge, this is one of the first studies conducted in Italy to investigate socioeconomic differences in EoL care among patients with cancer using routinely collected healthcare data. Its main strengths include the integration of individual- and area-level socioeconomic indicators, a large population-based cohort covering all cancer deaths in the Lazio Region over 4 years, and the use of multiple administrative data sources to assess several dimensions of care. This allowed for the analysis of EoL care patterns at the population level, minimizing selection bias and providing a broad representation of real-world EoL care across different healthcare settings.

However, the study has some limitations. First, it shares the constraints inherent to administrative healthcare data, which are not primarily collected to assess the quality or intensity of EoL care, and therefore provide only indirect measures that may be affected by residual confounding [18].

Second, the retrospective nature of the study could introduce biases; however, it is worth noting that previous research comparing retrospective and prospective approaches has revealed similar patterns of EoL care [10]. Third, information on cancer stage and date of diagnosis was unavailable, although both are important in shaping the intensity and organization of care delivered toward the end of life. Their absence limited our ability to fully account for clinical heterogeneity among patients. Future studies could address this limitation through linkage with regional cancer registry data, ideally integrating also information on Integrated Home Care and Palliative Care Networks in order to provide a more comprehensive picture of care delivered outside the hospital setting.

Information on performance status, prognosis, patients’ preferences, and family and caregiving support was also unavailable; this should be considered when interpreting the findings, as concordance with patients’ preferences could not be assessed and judgments regarding the appropriateness of EoL care are inherently shaped by individual values and goals of care. Finally, the cross-sectional nature of the study did not permit reconstruction of longitudinal care trajectories or examination of how healthcare use and service integration evolved during the EoL phase.

In conclusion, this study investigated socioeconomic differences in several indicators capturing different dimensions of EoL care among patients with cancer within a large regional healthcare system. Differences emerged in ED use, hospitalizations, in-hospital death, antineoplastic treatments, and outpatient opioid prescribing, suggesting that socioeconomic conditions may shape the organization and setting of care during the last phase of life. These results may provide useful insights for policymakers and healthcare planners to identify areas where inequalities in EoL care persist and where interventions aimed at improving equity and coordination of care may be needed. Incorporating a multidimensional set of EoL care indicators into regional or national monitoring systems, alongside longitudinal and clinically enriched data, could support a more comprehensive assessment of care and the development of more equitable EoL strategies.

## Supplementary Material

ckag148_Supplementary_Data

## Data Availability

The data that support the findings of this study are not publicly available and cannot be shared due to privacy restrictions.

## References

[1] Christensen K , Doblhammer G, Rau R et al Ageing populations: the challenges ahead. Lancet 2009;374:1196–208. 10.1016/S0140-6736(09)61460-4 19801098 PMC2810516

[2] OECD. *Time for Better Care at the End of Life, OECD Health Policy Studies*. Paris: OECD Publishing, 2023. 10.1787/722b927a-en

[3] Zimmermann C , Swami N, Krzyzanowska M et al Early palliative care for patients with advanced cancer: a cluster-randomised controlled trial. Lancet 2014;383:1721–30. 10.1016/S0140-673662416-2 24559581

[4] Temel JS , Greer JA, Muzikansky A et al Early palliative care for patients with metastatic non–small-cell lung cancer. N Engl J Med 2010;363:733–42. 10.1056/NEJMoa1000678 20818875

[5] Zaman M , Espinal-Arango S, Mohapatra A et al What would it take to die well? A systematic review of systematic reviews on the conditions for a good death. Lancet Healthy Longev 2021;2:e593–600. 10.1016/S2666-7568(21)00097-0 36098155

[6] Crawford GB , Dzierżanowski T, Hauser K et al ESMO Guidelines Committee. Care of the adult cancer patient at the end of life: ESMO clinical practice guidelines. ESMO Open 2021;6:100225. 10.1016/j.esmoop.2021.100225 34474810 PMC8411064

[7] Ferrell BR , Temel JS, Temin S et al Integration of palliative care into standard oncology care: American society of clinical oncology clinical practice guideline update. J Clin Oncol 2017;35:96–112. 10.1200/JCO.2016.70.1474 28034065

[8] Earle CC , Park ER, Lai B et al Identifying potential indicators of the quality of end-of-life cancer care from administrative data. J Clin Oncol 2003;21:1133–8. 10.1200/JCO.2003.03.059 12637481

[9] Ho TH , Barbera L, Saskin R et al Trends in the aggressiveness of end-of-life cancer care in the universal health care system of Ontario, Canada. J Clin Oncol 2011;29:1587–91. 10.1200/JCO.2010.31.9897 21402603 PMC3082976

[10] Setoguchi S , Earle CC, Glynn R et al Comparison of prospective and retrospective indicators of the quality of end-of-life cancer care. Journal of Clinical Oncology 2008;26:5671–8. 10.1200/JCO.2008.16.3956 19001333 PMC2650390

[11] Bekelman JE , Halpern SD, Blankart CR et al International Consortium for End-of-Life Research (ICELR). Comparison of site of death, health care utilization, and hospital expenditures for patients dying with cancer in 7 developed countries. JAMA 2016;315:272–83. 10.1001/jama.2015.18603 26784775

[12] De Palma R , Fortuna D, Hegarty SE et al Effectiveness of palliative care services: a population-based study of end-of-life care for cancer patients. Palliat Med 2018;32:1344–52. 10.1177/0269216318778729 29886795

[13] Formoso G , Marino M, Guberti M et al End-of-life care in cancer patients: how much drug therapy and how much palliative care? Record linkage study in Northern Italy. BMJ Open 2022;12:e057437. 10.1136/bmjopen-2021-057437 PMC9083387 35523497

[14] Marmot M. Health equity in England: the Marmot review 10 years on. BMJ 2020;368:m693. 10.1136/bmj.m693 32094110

[15] Vineis P , Avendano-Pabon M, Barros H et al Special report: the biology of inequalities in health: the lifepath consortium. Front Public Health 2020;8:118. 10.3389/fpubh.2020.00118 32478023 PMC7235337

[16] Li J , Li J, Xu X et al Social determinants of health, accelerated biological aging, and long-term health outcomes. Nat Commun 2025;17:900. 10.1038/s41467-025-67622-7 41444248 PMC12830646

[17] Davies JM , Maddocks M, Chua KC et al Socioeconomic position and use of hospital-based care towards the end of life: a mediation analysis using the English longitudinal study of ageing. Lancet Public Health 2021;6:e155–63. 10.1016/S2468-2667(20)30292-9 33571459 PMC7910274

[18] Bainbridge D , Seow H. Measuring the quality of palliative care at end of life: an overview of data sources. Healthy Aging Clin Care Elder 2016;8:9–15. 10.4137/hacce.s18556

[19] Davies JM , Sleeman KE, Leniz J et al Socioeconomic position and use of healthcare in the last year of life: a systematic review and meta-analysis. PLoS Med 2019;16:e1002782. 10.1371/journal.pmed.1002782 31013279 PMC6478269

[20] Rosano A , Pacelli B, Zengarini N et al Update and review of the 2011 Italian deprivation index calculated at the census section level. Epidemiol Prev 2020;44:162–70. 10.19191/EP20.2-3.P162.039 32631016

[21] Giannubilo I , Battistuzzi L, Blondeaux E et al Patterns of care at the end of life: a retrospective study of Italian patients with advanced breast cancer. BMC Palliat Care 2024;23:129. 10.1186/s12904-024-01460-0 38778303 PMC11110270

[22] Chiaruttini MV , Corli O, Pizzuto M et al Palliative medicine favourably influences end-of-life cancer care intensity: a large retrospective database study. BMJ Support Palliat Care 2022;14:e1293–301. 10.1136/spcare-2022-004050 36522144

[23] Charlson ME , Pompei P, Ales KL et al A new method of classifying prognostic comorbidity in longitudinal studies: development and validation. J Chronic Dis 1987;40:373–83. 10.1016/0021-9681(87)90171-8 3558716

[24] Davey Smith G , Hart C, Hole D et al Education and occupational social class: which is the more important indicator of mortality risk? J Epidemiol Community Health (1978) 1998;52:153–60. 10.1136/jech.52.3.153 PMC1756692 9616419

[25] Lindberg MH , Chen G, Olsen JA et al Combining education and income into a socioeconomic position score for use in studies of health inequalities. BMC Public Health 2022;22:969. 10.1186/s12889-022-13366-8 35562797 PMC9107133

[26] Earle CC , Neville BA, Landrum MB et al Evaluating claims-based indicators of the intensity of end-of-life cancer care. Int J Qual Health Care 2005;17:505–9. 10.1093/intqhc/mzi061 15985505

[27] Grunfeld E , Lethbridge L, Dewar R et al Towards using administrative databases to measure population-based indicators of quality of end-of-life care: testing the methodology. Palliat Med 2006;20:769–77. 10.1177/0269216306072553 17148531 PMC3741158

[28] Colombet I , Bouleuc C, Piolot A et al EFIQUAVIE Study Group. Multicentre analysis of intensity of care at the end-of-life in patients with advanced cancer, combining health administrative data with hospital records: variations in practice call for routine quality evaluation. BMC Palliat Care 2019;18:35. 10.1186/s12904-019-0419-4 30953487 PMC6451228

[29] World Health Organization*. WHO Guidelines for the Pharmacological and Radiotherapeutic Management of Cancer Pain in Adults and Adolescents*. Geneva, Switzerland: World Health Organization, 2019. https://www.who.int/publications/i/item/9789241550390 30776210

[30] Gomes B , Higginson IJ, Calanzani N et al PRISMA. Preferences for place of death if faced with advanced cancer: a population survey in England, Flanders, Germany, Italy, The Netherlands, Portugal and Spain. Ann Oncol 2012;23:2006–15. 10.1093/annonc/mdr602 22345118

[31] Henson LA , Gao W, Higginson IJ et al Emergency department attendance by patients with cancer in their last month of life: a systematic review and meta-analysis. J Clin Oncol 2015;33:370–6. 10.1200/JCO.2014.57.3568 25534384

[32] Seow H , Barbera L, Howell D et al Using more end-of-life homecare services is associated with using fewer acute care services: a population-based cohort study. Med Care 2010;48:118–24. 10.1097/MLR.0B013E3181C162EF 20057327

[33] Walsh B , Laudicella M. Disparities in cancer care and costs at the end of life: evidence from England’s national health service. Health Aff 2017;36:1218–26. 10.1377/hlthaff.2017.0167 28679808

[34] Chen H , Walabyeki J, Johnson M et al An integrated understanding of the complex drivers of emergency presentations and admissions in cancer patients: qualitative modelling of secondary-care health professionals’ experiences and views. PLoS One 2019;14:e0216430. 10.1371/JOURNAL.PONE.0216430 31048875 PMC6497383

[35] Leniz J , Henson LA, Potter J et al Association of primary and community care services with emergency visits and hospital admissions at the end of life in people with cancer: a retrospective cohort study. BMJ Open 2022;12:e054281. 10.1136/BMJOPEN-2021-054281 PMC8867349 35197345

[36] Cenzer I , Covinsky KE, Cross SH et al Wealth disparities in end-of-life symptom burden among older adults. JAMA Netw Open 2025;8:e250201. 10.1001/JAMANETWORKOPEN.2025.0201 40048165 PMC11886723

[37] Cristofalo A , Cascini S, Cesaroni G et al Educational disparities in dementia incidence and healthcare utilization: evidence from a cohort study in Italy. Soc Sci Med 2025;380:118233. 10.1016/j.socscimed.2025.118233 40440743

[38] Hanlon P , Politis M, Wightman H et al Frailty and socioeconomic position: a systematic review of observational studies. Ageing Res Rev 2024;100:102420. 10.1016/j.arr.2024.102420 39025269

[39] Bowers SP , Chin M, O’Riordan M et al The end of life experiences of people living with socio-economic deprivation in the developed world: an integrative review. BMC Palliat Care 2022;21:193. 10.1186/s12904-022-01080-6 36335335 PMC9636719

[40] French M , Keegan T, Anestis E et al Exploring socioeconomic inequities in access to palliative and end-of-life care in the UK: a narrative synthesis. BMC Palliat Care 2021;20:179. 10.1186/s12904-021-00878-0 34802450 PMC8606060

