# Supplementary material for: Socioeconomic differences in the intensity of end-of-life care among patients with cancer: a population-based study in the Lazio region, Italy
Source: Eur J Public Health. 2026 Aug 17;36(5):ckag148. doi: 10.1093/eurpub/ckag148 (PMC13479746; doi:10.1093/eurpub/ckag148)
Supplement: Supplementary file 1 [file ckag148_supplementary_data.zip › Supplementary_References.docx]

**Supplementary References**

[S41]	Gomes B, Higginson IJ. Factors influencing death at home in terminally ill patients with cancer: systematic review. BMJ 2006;332:515–21. https://doi.org/10.1136/bmj.38740.614954.55.

[S42]	Costa V. The Determinants of Place of Death: An Evidence-Based Analysis. Ont Health Technol Assess Ser 2014;14:1–78.
