# Supplementary material for: Socioeconomic differences in the intensity of end-of-life care among patients with cancer: a population-based study in the Lazio region, Italy
Source: Eur J Public Health. 2026 Aug 17;36(5):ckag148. doi: 10.1093/eurpub/ckag148 (PMC13479746; doi:10.1093/eurpub/ckag148)
Supplement: Supplementary file 1 [file ckag148_supplementary_data.zip › Table_S1.docx]

**Table S1.** Codes used to identify antineoplastic treatments, opioid use and life-sustaining procedures in administrative healthcare databases

| **Indicator** | **Treatment/procedure** | **ICD-9-CM procedure codes** | **DRG codes** | **Outpatient specialist care codes** | **ATC codes** |
| --- | --- | --- | --- | --- | --- |
| Antineoplastic treatment | Chemotherapy | 99.25 | 410 | 99.25; 99.25.01; 90.20.5 | L01 |
| Antineoplastic treatment | Immunotherapy | 99.28 | — | 92.28.4; 92.28.5 | — |
| Opioid use | Opioid analgesics | — | — | — | N02A |
| Radiotherapy | Radiotherapy | 92.24; 92.25; 92.26; 92.29 | — | 92.24.1–92.24.4; 92.21.1; 92.23.1–92.23.3; 92.25.1–92.25.2; 92.27.1; 92.27.3; 92.27.4; 92.27.5 | — |
| Life-sustaining treatments/procedures | Assisted ventilation | 96.04 | — | — | — |
| Life-sustaining treatments/procedures | Parenteral/enteral nutrition | 43.1; 43.11; 43.19; 44.32; 46.32; 46.39 | — | — | — |
| Life-sustaining treatments/procedures | Cardiopulmonary resuscitation | 93.93; 99.60; 99.62; 99.63; 37.91; 37.92 | — | — | — |

**Note:** ICD-9-CM, International Classification of Diseases, Ninth Revision, Clinical Modification; DRG, Diagnosis-Related Group; ATC, Anatomical Therapeutic Chemical classification system. Outpatient specialist care codes refer to procedure codes recorded in the Italian outpatient specialist care database.
