# Supplementary material for: Socioeconomic differences in the intensity of end-of-life care among patients with cancer: a population-based study in the Lazio region, Italy
Source: Eur J Public Health. 2026 Aug 17;36(5):ckag148. doi: 10.1093/eurpub/ckag148 (PMC13479746; doi:10.1093/eurpub/ckag148)
Supplement: Supplementary file 1 [file ckag148_supplementary_data.zip › Table_S2.docx]

**Table S2** Characteristics of the cohort by Area-based SEP index

|  | **Socioeconomic Position (Area-based index)** | | | | |
| --- | --- | --- | --- | --- | --- |
|  | Low | Medium-Low | Medium | Medium High | High |
| *Overall (n)* | 12104 | 17420 | 16188 | 16087 | 15186 |
| **Sex** |  |  |  |  |  |
| *M* | 52,7% | 53,7% | 55,2% | 56,0% | 55,7% |
| *F* | 47,3% | 46,3% | 44,8% | 44,0% | 44,3% |
| **Age** |  |  |  |  |  |
| *35-64* | 15,40% | 17,10% | 19,6% | 20,6% | 21,5% |
| *65-74* | 22,3% | 23,5% | 24,2% | 24,8% | 24,4% |
| *75+* | 62,4% | 59,5% | 56,2% | 54,6% | 54,1% |
| *median* | 76 | 76 | 75 | 74 | 74,00 |
| *1° quartile-3° quartile* | 69-85 | 69-85 | 67-84 | 67-83 | 66-83 |
| **N of comorbidities** |  |  |  |  |  |
| *0-1* | 27,7% | 26,2% | 24,7% | 23,7% | 21,8% |
| *2* | 37,1% | 36,5% | 36,8% | 36,1% | 35,8% |
| *3* | 19,8% | 19,7% | 20,1% | 20,7% | 20,9% |
| *4+* | 15,4% | 17,6% | 18,4% | 19,5% | 21,5% |
| **Tumor site** |  |  |  |  |  |
| *Lung bronchi and trachea* | 22,1% | 21,2% | 21,3% | 22,8% | 23,9% |
| *Upper gastrointestinal tract* | 4,8% | 5,4% | 5,6% | 6,0% | 5,8% |
| *Colon and rectum* | 10,9% | 11,4% | 12,0% | 11,1% | 11,0% |
| *Liver and biliary tract* | 5,4% | 5,4% | 5,7% | 6,1% | 6,1% |
| *Pancreas* | 7,1% | 7,4% | 7,0% | 6,4% | 6,5% |
| *Breast* | 7,6% | 7,0% | 6,4% | 6,6% | 6,5% |
| *Female genital system* | 4,2% | 3,9% | 4,1% | 4,2% | 3,9% |
| *Male genitourinary system* | 4,8% | 4,6% | 4,5% | 4,3% | 3,8% |
| *Kidney and urinary tract* | 6,2% | 6,1% | 6,5% | 5,9% | 6,3% |
| *Central nervous system* | 3,3% | 3,5% | 3,4% | 3,2% | 3,2% |
| *Other solid tumors* | 5,6% | 5,9% | 5,8% | 5,9% | 5,8% |
| *Lymphatic and hematopoietic tissue tumors* | 18,0% | 18,3% | 17,7% | 17,7% | 17,2% |
| **LHA of residence** |  |  |  |  |  |
| *LHA of Rome 1* | 37,1% | 24,7% | 16,2% | 12,5% | 15,5% |
| *LHA of Rome 2* | 27,9% | 28,4% | 19,8% | 19,8% | 28,6% |
| *LHA of Rome 3* | 11,7% | 14,4% | 11,8% | 8,2% | 11,2% |
| *LHA of Rome 4* | 3,2% | 3,5% | 6,6% | 6,9% | 4,5% |
| *LHA of Rome 5* | 1,2% | 3,8% | 9,1% | 13,8% | 7,5% |
| *LHA of Rome 6* | 3,3% | 5,1% | 9,0% | 10,4% | 9,4% |
| *LHA of Viterbo* | 4,7% | 5,7% | 6,6% | 5,8% | 3,2% |
| *LHA of Rieti* | 3,3% | 3,1% | 2,8% | 2,5% | 1,5% |
| *LHA of Latina* | 3,3% | 4,2% | 7,9% | 11,8% | 12,1% |
| *LHA of Frosinone* | 4,4% | 7,2% | 10,1% | 8,2% | 6,5% |
| *** missing values were not reported* |  |  |  |  |  |

**Note:** LHA, Local Health Authority. Area-based SEP index data were missing for 7,951 individuals
